# Opportunities to address the failure of online food retailers to ensure access to required food labelling information in the USA

**DOI:** 10.1017/S1368980021004638

**Published:** 2022-05

**Authors:** Jennifer L Pomeranz, Sean B Cash, Morgan Springer, Inés M Del Giudice, Dariush Mozaffarian

**Affiliations:** 1 School of Global Public Health, New York University, New York, NY, USA; 2 Friedman School of Nutrition Science and Policy, Tufts University, Boston, MA, USA

**Keywords:** Online food retail, Food labelling, Federal regulatory agencies, Supplemental Nutrition Assistance Programme

## Abstract

**Objective:**

The rapid growth in web-based grocery food purchasing has outpaced federal regulatory attention to the online provision of nutrition and allergen information historically required on food product labels. We sought to characterise the extent and variability that online retailers disclose required and regulated information and identify the legal authorities for the federal government to require online food retailers to disclose such information.

**Design:**

We performed a limited scan of ten products across nine national online retailers and conducted legal research using LexisNexis to analyse federal regulatory agencies’ authorities.

**Setting:**

USA.

**Participants:**

N/A.

**Results:**

The scan of products revealed that required information (Nutrition Facts Panels, ingredient lists, common food allergens and per cent juice for fruit drinks) was present, conspicuous and legible for an average of only 36·5 % of the products surveyed, ranging from 11·4 % for potential allergens to 54·2 % for ingredients lists. More commonly, voluntary nutrition-related claims were prominently and conspicuously displayed (63·5 % across retailers and products). Our legal examination found that the Food and Drug Administration, Federal Trade Commission and United States Department of Agriculture have existing regulatory authority over labelling, online sales and advertising, and Supplemental Nutrition Assistance Programme retailers that can be utilised to address deficiencies in the provision of required information in the online food retail environment.

**Conclusions:**

Information regularly provided to consumers in conventional settings is not being uniformly provided online. Congress or the federal agencies can require online food retailers disclose required nutrition and allergen information to support health, nutrition, equity and informed consumer decision-making.

Good nutrition plays a foundational role in the prevention and treatment of health conditions, including CHD, stroke, type 2 diabetes, obesity and certain forms of cancer^(1)^. People with diet-related conditions and those who are older and have children regularly review nutrition, ingredient and health-related statements on food labelling^(2)^. Thus, the rapidly growing online food retail environment, where nutrition information is not consistently provided^(3)^, is of increasing concern for the nutrition of consumers overall and for health equity^(4)^.

In 2014, Congress instructed the United States Department of Agriculture (USDA) to pilot online purchasing for the Supplemental Nutrition Assistance Program (SNAP), which provides supplementary income to low-income participants to purchase food. In 2019, the USDA launched the pilot programme with eight retailers^(5)^. As of April 2021, forty-seven states and the District of Columbia were participating in the online food purchasing pilot for SNAP participants^(5)^. Although there was initial hesitancy for online food purchasing for SNAP participants^(6)^, the COVID-19 pandemic prompted a dramatic change in all US consumer habits.

Between 2019 and 2020, consumers’ use of online platforms to purchase at least some of their groceries rose from 19 % to 79 %^(7,8)^, and this number is expected to grow^(9,10)^. Forecasts expect online grocery orders to make up 21·5 % of total US grocery sales by just 2023^(9)^. These seismic shifts have outpaced regulatory attention to the online provision of nutritional and allergen information required to be disclosed on food product labels in the USA. One assumption of food labelling regulations is that consumers are able to inspect food packaging to access required information to make informed purchasing decisions^(11)^. Historically, retailers were not responsible for providing this information, as it is required on product labels. The online setting thus raises questions of which entity along the supply chain is responsible for ensuring that consumers can access required product label information and which regulatory agency has authority to require it.

Although Congress can pass a new law or amend existing legislation to mandate that required information is provided online, three federal agencies have authority that may be leveraged to address the issue now: the Food and Drug Administration (FDA), with authority over food labelling^(12)^; Federal Trade Commission (FTC), with authority over food advertising and online sales^(13,14)^; and USDA which oversees SNAP.

Research conducted prior to the pandemic found that twelve online food retailers did not consistently disclose required nutrition information^(3)^. The extent that online food retailers currently display required food labelling information on their web pages and the range of regulatory solutions for the federal agencies to require the disclosure of required food labelling information in the online marketplace are not established. To address these knowledge gaps, this study first performed a limited scan of ten products across nine national online retailers to identify the provision of required and regulated information on product labels to inform the second aim, which was to research and examine the legal authorities and limitations of the federal government to require the disclosure of mandated food labelling information on online food retail platforms.

## Methods

This research focused on packaged foods regulated by the FDA, which are required to have a standardised information panel that discloses the Nutrition Facts Panel, ingredient list, common food allergens and, for fruit drinks, the per cent juice^(12)^. Although the front of the package (i.e. the principal display panel) is also regulated, manufacturers primarily use this panel for branding and advertising, including the display of health and nutrition-related claims. Although the scan captured these claims, the legal analysis did not include an evaluation of the regulatory agencies’ authorities related to the principal display panel.

### Scan of product information available online

To investigate the range of information provided through online food retailers, our team captured information from the web pages for ten major national packaged products across nine retailers. This research was not intended to be exhaustive but rather sought to provide a sample for descriptive analysis and to inform the subsequent legal analysis. The products were chosen prior to collection of any product information data from retailer web pages based on three product categories most purchased by SNAP participants (bread, cereal and drinks)^(15)^ and brands most sold within those product categories^(16,17)^. We over-selected cereals and drinks to be able to compare findings across similar products and product types. The products included Wonder Bread Classic White Bread (Flowers Foods, Inc.), Cheerios (General Mills), Honey Nut Cheerios (General Mills), Honey Bunches of Oats with Almonds (Post Holdings), Frosted Flakes (Kellogg’s Company), Coca-Cola two-litre bottles (Coca-Cola Company), Kool-Aid Jammers Cherry pouches (Kraft Foods, Inc.) and three Capri Sun products (Kraft Heinz): Capri Sun Fruit Punch pouches, Capri Sun Roarin’ Waters Fruit Punch Wave pouches and Capri Sun Organic Fruit Punch pouches. These ten products were accessed, viewed and coded from nine online retailers – the eight retailers involved in the initial launch of the SNAP Online Purchasing Pilot (Amazon, Dash’s Market, Fresh Direct, Hy-Vee, Inc., Safeway, ShopRite [via Instacart], Walmart Stores Inc., and Wright’s Markets, Inc.^(5)^) plus Stop and Shop, a prominent supermarket chain that uses the Peapod online platform and which has historically been a major player in online grocery retail^(18)^.

In January–February 2021, using a computer, a research assistant accessed the product and brand that matched the description above according to the first one displayed from each retailer and took screenshots of the full product pages (expanded through clicks, hovering and scrolling); the researcher cleared cookies and history between each product and retailer to prevent ordering effects. Two researchers then separately coded each product across all retailers for the presence, location and legibility of six informational items: (1) the Nutrition Facts Label; (2) ingredients list; (3) common allergens for cereals and bread; (4) the per cent juice for fruit drinks; and health or nutrition-related claims (5) provided on the product packaging image or (6) separately in the text of the product description on the web page. For analysis purposes, each item was considered ‘Present, Conspicuous and Legible’ if it existed on the product web page, could be accessed without clicking or scrolling, and the information could be read by the coders without modifying the image (e.g. through magnification); we calculated the averages for each information category across the nine retailers. We also identified the percentage of items entirely not present for each product across these retailers. The percentage of products not falling into these two categories therefore represents the number of products for which an attribute was present but not conspicuousness or legible across retailers. Finally, we calculated grand averages for both the number of products for which each information was both ‘present, conspicuous and legible’ and ‘not present’ as the mean percentages across all stores and products.

### Legal landscape

To identify existing authorities for the FDA, FTC and USDA to regulate online food retail labelling without new Congressional legislation, using LexisNexis in February–April 2021, we examined the United States Code (federal statutes) and Code of Federal Regulations associated with labelling, online sales, food retailers and SNAP retailers, and relevant case law associated with these code sections. Although Congress has the authority to enact new legislation or amend existing laws, a search of Congress.gov indicated that no such legislation has been introduced, at least since 2019.

## Results

### Online retail provision of information

The results of our scan of regulated information through online retail platforms are shown in Table 1. The required Nutrition Facts Panel was present, conspicuous and legible for 45·7 % of the ten products across retailers (ranging from 11·1 % to 80·0 % by product) and the ingredient list was present, conspicuous, and legible for 54·2 % of all observations (ranging from 33·3 to 80·0 %). These two required disclosures were not present at all for almost 11 % of products across retailers. As noted in Table 1, there were also differences across similar products from the same manufacturer: Honey Nut Cheerios *v*. regular Cheerios and Capri Sun drinks, which contain varying amounts and types of added sweeteners and juice^(19)^. The least consistently disclosed required information was common food allergens, not present for 63·5 % of products that contain common allergens (22·2–87·5 %), followed by required per cent juice which was not present for 38·3 % of the fruit drinks (22–62·5 %). Overall, required information was present, conspicuous and legible an average of only 36·5 % of the time across these four mandatory information categories for all products surveyed.

**Table 1:** Percentage of product information items displayed and readily available for ten products across nine major US online retailers in January–February 2021

	Nutrition facts panel		Ingredients list		Potential allergens		% Juice		Claims on product package image		Product claims in web page text	
Product (*n*)	Present, conspicuous and legible	Not present	Present, conspicuous and legible	Not present	Present, conspicuous and legible	Not present	Present, conspicuous and legible	Not present	Present, conspicuous and legible	Not present	Present, conspicuous and legible	Not present
Capri sun fruit punch pouches (*n* 9)	66·7 %	0·0 %	55·6 %	11·1 %	n/a	n/a	44·4 %	22·2 %	88·9 %	0·0 %	55·6 %	22·2 %
Capri Sun Organic Fruit Punch Pouches (*n* 5)	80·0 %	0·0 %	80·0 %	0·0 %	n/a	n/a	40·0 %	40·0 %	80·0 %	0·0 %	40·0 %	20·0 %
Capri Sun Roarin’ Waters Pouches (*n* 7)	57·1 %	14·3 %	71·4 %	14·3 %	n/a	n/a	28·6 %	28·6 %	71·4 %	0·0 %	42·9 %	28·6 %
Kool-Aid Jammers Cherry Pouches (*n* 8)	37·5 %	37·5 %	50·0 %	25·0 %	n/a	n/a	25·0 %	62·5 %	100·0 %	0·0 %	50·0 %	25·0 %
Coca-Cola (*n* 9)	66·7 %	0·0 %	55·6 %	11·1 %	n/a	n/a	n/a	n/a	0·0 %	100·0 %	12·5 %	87·5 %
Cheerios (*n* 9)	33·3 %	0·0 %	44·4 %	11·1 %	0·0 %	100·0 %*	n/a	n/a	88·9 %	0·0 %	50·0 %	12·5 %
Honey Nut Cheerios (*n* 9)	11·1 %	33·3 %	33·3 %	11·1 %	11·1 %	66·7 %	n/a	n/a	88·9 %	0·0 %	37·5 %	25·0 %
Honey Bunches of Oats, With Almonds (*n* 9)	33·3 %	0·0 %	44·4 %	11·1 %	33·3 %	22·2 %	n/a	n/a	66·7 %	22·2 %	25·0 %	25·0 %
Kellogs Frosted Flakes (*n* 9)	33·3 %	11·1 %	44·4 %	11·1 %	0·0 %	77·8 %	n/a	n/a	0·0 %	66·7 %	25·0 %	50·0 %
Wonder Bread (*n* 8)	37·5 %	12·5 %	62·5 %	0·0 %	12·5 %	87·5 %	n/a	n/a	50·0 %	25·0 %	50·0 %	50·0 %
Average across all products (sd)	45·7 %	10·9 %	54·2 %	10·6 %	11·4 %	70·8 %	34·5 %	38·3 %	63·5 %	21·4 %	38·8 %	34·6 %

* Cheerios contains no common food allergens but is in a category where potential food allergens are common so was included in the analysis. The average total Not Present for Food Allergens excluding Cheerios is 63·5 %.

Notes: values represent percentages of each product from the nine major online retailers; the number of retailers that sold each product is indicated in parentheses after each product name. ‘Present, Conspicuous and Legible’ refers to the information being present and visible without additional clicking or scrolling and without legibility concerns (e.g. small print, blurriness). Percentages exclude products where the claim is inapplicable (i.e. ‘% juice’ for non-fruit drinks or potential allergens for product categories without any common food allergen ingredients, marked n/a) or where an item was missing.

In contrast, voluntary health and nutrition-related claims on the product package image were present, conspicuous and legible across 63·5 % of retailers and products. Online retailers also displayed such claims directly in the web page texts themselves across 38·8 % of products, for example, Coca-Cola had a ‘low sodium’ claim on one website – something not found on its packaging.

### Legal assessment

This research identified existing authorities that may directly apply to online food retail or could serve as models for future regulation, as set forth in Table 2.

**Table 2:** US federal agencies’ regulatory authority to require food retailers disclose mandated food labelling information prominently and conspicuously online

Agency	Authority for online retail	Interpretation	Potential regulatory actions
Food and Drug Administration (FDA)	Labelling includes all written, printed or graphic matter accompanying an article at any time while such article is in interstate commerce or held for sale after shipment or delivery in interstate commerce ^(20)^.	The broad definition of labelling should be interpreted to include the display of products online.	The FDA may be able to use existing authority to engage in rulemaking to explicitly require the provision of required nutrition information be highlighted for prominent and conspicuous viewing online.
	Advertising which performs the same function as labelling is under the purview of the FDA^(23)^.		
	FDA regulations require the information panel must be prominently placed and with such conspicuousness as to render it likely to be read and understood by the ordinary individual under customary conditions of purchase and use^(12,24)^.	The display of products online also should be considered a customary condition of purchase, meaning required information should be prominently and conspicuously displayed.	FDA could engage in enforcement actions against online retailers who fail to disclose required nutrition labelling.
	Congress granted the FDA the authority to highlight required information on labelling, if it determines that such highlighting will assist consumers in maintaining healthy dietary practices^(11,12)^.		FDA could issue a guidance document to clarify its interpretation of labelling to include online food retail and recommend online food retailers meets current regulatory requirements for food labelling.
Federal Trade Commission (FTC)	FTC has authority to address unfair and deceptive acts and practices with respect to the food advertising, advertising claims, marketing and promotional activities, mail order advertising and sales practices, including sales online^(25–28)^.	It is arguably the case that all missing or difficult to find required information is an unfair and deceptive act or practice for online food retailers, based on consumers’ inability to identify this information and the broad potential for consumers to choose differently if they had this information. Further, there is a real potential for harm for the failure to disclose certain information like common food allergens and ingredients such as Na and sugar for particular consumers.	The FTC could engage in enforcement actions against food retailers that fail to provide mandatory nutrition information as an unfair and deceptive act or practice.
	FTC has authority over retail food stores in relation to accurate representations of stock and availability in advertisements^(34.35)^.	Retail food regulations highlight FTC’s authority to oversee misrepresentations related to advertising by food retailers.	The FTC could issue a guidance document to ensure online food retailers’ display of products is not unfair or deceptive.
			Congress would need to provide FTC authority under the Administrative Procedures Act for it to meaningfully engage in timely rulemaking on the topic.
United States Department of Agriculture (USDA)	The USDA administers federal food programmes, most notably the Supplemental Nutrition Assistance Programme (SNAP) and has the authority to designate which retail food stores are authorised to accept and redeem SNAP benefits, including online entities that sell food^(36,37,41)^.	The USDA has the regulatory authority to identify factors for consideration to determine whether retail applicants qualify to accept and redeem SNAP benefits. It may designate the provision of required nutrition and allergen information as one such prerequisite. Consumer access to required nutrition information effectuates the purpose of the programme.	USDA should revise regulations related to retail food stores to require online retail platforms that accept and redeem SNAP benefits to ensure all required labelling is immediately visible, and conspicuously and legibly displayed.
	USDA has the authority to issue regulations to provide for the submission of applications for approval of SNAP retail food stores and ‘for the approval of those applicants whose participation will effectuate the purposes’ of SNAP^(37)^. SNAP’s Declaration of Policy includes ‘raising levels of nutrition among low-income households^(38)^.’		

The FDA has authority over ‘labels’ and ‘labelling’ which are ‘written, printed or graphic matter’ ‘upon the immediate container’ or ‘accompanying’ the product, respectively^(20)^. The FDA and US courts have consistently interpreted the term ‘labelling’ broadly, to go beyond labels and shelf tags to include ‘all written, printed, or graphic matter accompanying an article at any time while such article is in interstate commerce or held for sale after shipment or delivery in interstate commerce^(21,22)^’.

A seminal Supreme Court case on FDA’s authority over ‘labelling’ is *Kordel v. USA* ^(23)^. The FDA enforces provisions against misbranding for food and drugs, among other products. In *Kordel v. USA*., a drug company’s product was found to be misbranded even though the statements in question were on pamphlets shipped to consumers separate from the drugs themselves. The Court found the products and pamphlets were ‘interdependent’ because the pamphlets supplemented or explained the products^(23)^. The Court stated that labelling requirements could not be circumvented by designating information as ‘advertising’ when it ‘performs the same function’ as it would if it were on the article, container or wrapper^(23)^. Therefore, the Court found that ‘advertising which performs the function of labelling’ is under the purview of the FDA. It is thus possible that since the display of food products online performs the same function as labelling for online consumers, online food sales may be under the purview of the FDA even if it is also considered advertising. If this is the case, online food retailers could not circumvent FDA’s labelling regulations.

In addition to proscribing information required to be displayed on food labels, the FDA ensures required information is ‘prominently placed’ and ‘with such conspicuousness’ ‘as to render it likely to be read and understood’ by consumers ‘under customary conditions of purchase and use^(12,24)^’. The ‘customary conditions of purchase’ now seems to include online food sale through retailer web pages; in this case, the prominent and conspicuous requirement for mandatory nutritional information may be considered to apply to the online display of food products for sale.

The FDA also has the authority to update certain regulations^(11)^ and the agency used this authority in 2016, to revise the Nutrition Facts Panel. Another clause of the same provision permits the FDA to pass regulations to require any required information on labelling ‘to be highlighted’ with ‘larger type, bold type or contrasting colour’ if it ‘determines that such highlighting will assist consumers in maintaining healthy dietary practices^(12)^’. This may be interpreted as authority for the FDA to engage in rulemaking to highlight required information online to address deficiencies and support healthy dietary practices.

Turning to the FTC, the agency has consumer protection authority to address false, deceptive and unfair acts or practices related to food advertising, marketing, promotion, mail order and online sales^(25,26)^. Unfair is defined as an act or practice that ‘causes or is likely to cause substantial injury to consumers which is not reasonably avoidable by consumers themselves and not outweighed by countervailing benefits to consumers or to competition^(27)^’. Retailers’ failure to display required information and the inconsistency across products and retailers may render it unclear to consumers that such information is missing, making the deficiency unavoidable. This is especially concerning for consumers who have limited options for online delivery due to their location (e.g. rural area) or if they are SNAP participants using an approved SNAP retailer. There is also a potential for health harm for failure to disclose common food allergens and other ingredients of concern such as Na, making these deficiencies likely to cause injury.

Deception is defined as a ‘representation, omission or practice’ that is ‘material’ and likely to mislead a consumer, ‘analysed from the perspective of a consumer acting reasonably in the circumstances^(28)^’. Materiality can be found if the act or practice is likely to affect consumers’ decision with respect to the product, meaning they would have ‘chosen differently but for the deception^(28)^’. Retailers’ failure to provide required information may be deemed a material omission because it is likely to mislead consumers to purchase products they may not have otherwise. Reasonable consumers may not have purchased the food online or the specific food they purchased, but for the lack of required information.

Congress has also granted the FTC authority to prescribe ‘interpretive rules and general statements of policy^(29)^’, but rulemaking under the FTC Act takes years to complete^(30)^. Nonetheless, FTC has rules that provide precedent for the regulation of required information for online food retailers. For example, wool products are considered ‘falsely or deceptively advertised in any mail order promotional material’ unless the ‘product description states in a clear and conspicuous manner’ the country of origin^(31)^. The FTC thus requires clear and conspicuous country of origin information in mail order catalogues and promotional material^(32)^, defined as ‘any materials, used in the direct sale or direct offering for sale’, disseminated in print *or electronically* to consumers to solicit purchase ‘*without examining the actual product purchased* ^(33)^’, – just like for online food retail. The FTC also has limited regulatory authority over retail food stores in relation to stores’ accurate representations of stock and availability in advertisements^(34,35)^. This regulation further highlights FTC’s authority for overseeing misrepresentations related to advertising by food retailers.

Lastly, we identified regulatory authority of the USDA over retail food stores. Congress granted the USDA discretionary authority to identify factors for consideration and designate which stores are authorised to accept and redeem SNAP benefits^(36,37)^. As such, the USDA has the authority to issue regulations related to the approval of SNAP retail food stores, including those ‘whose participation will effectuate the purposes’ of SNAP^(37)^, one of which is to raise nutrition levels of participants^(38)^. For example, although Congress designated the stocking requirements for retail food stores to qualify to accept SNAP (i.e. the number of foods in each staple and perishable food category)^(39)^, the USDA used its discretion to further alter eligibility criteria (e.g. the depth of stocking required)^(40)^. Congress therefore envisioned that the USDA would have the regulatory authority to require SNAP retailers engage in additional practices to further the goals of the programme. Moreover, Congress amended the definition of SNAP ‘retail food store’ to include an ‘online entity that sells food’ in 2018^(41)^. Thus, the USDA appears to have the authority to require online retailers, as a prerequisite to qualifying as SNAP retailers, to prominently, conspicuously and legibly display required information to support the nutritional purpose of the programme.

## Discussion

The online food retail environment is of increasing relevance for influencing nutrition and health. This is true for both overall and health equity, given the expansion of online SNAP sales to almost every state in the nation. US federal regulations require specific nutrition, ingredient and allergen information to be disclosed on food labels with precise size, location and legibility requirements to protect consumers and create consistency across products and brands. However, our study identified that even with a limited scan of ten popular products across nine major retailers, there was inadequate disclosure of such information to consumers in online environments. Similar to a study conducted in 2018 which assessed the availability, accessibility and legibility of nutrition information for 26 products across 12 online retailers, we likewise found that the required Nutrition Facts Panel and ingredient information were not universally available for packaged food items and this information was not always easily accessed or legible^(3)^. We also found differences in the provision of required information even among similar products. Notably, in our study, allergen warnings were the least consistently provided in the online retail environments, rendering it potentially unclear whether the information is missing (e.g. Honey Nut Cheerios) or not required due to lack of a common allergen (Cheerios). In addition, we found that health and nutrition-related claims, which are used to promote products, were most commonly provided conspicuously and legibly, and retailers’ web pages provided an additional location to display such claims.

Our findings raise several key questions for future research. Why is nutritional information not sufficiently available, and why do differences exist across both products and retailers? How does lack of legally mandated information influence consumer purchasing decisions? Does the variation by product and retailer lead to consumer confusion to determine whether key information (e.g. allergens) is missing or not applicable to that specific product? And, are these deficiencies and differences merely random waypoints in a still-evolving online landscape, or do they reflect strategic differences in how companies wish to present different products (e.g. perhaps failing to clearly show information for less healthy products) or distinguish themselves from competitors?

Congress enacted the Fair Packaging and Labelling Act based on the rationale that informed consumers ‘are essential to the fair and efficient functioning of a free market economy^(11)^’. What has fundamentally changed is not the rationale, necessity or requirements for mandatory labelling, but rather the effective regulated party: the retailer, rather than the manufacturer.

Our legal research reveals that each of the three federal agencies evaluated likely have the authority to address the online provision of nutritional information in their own way. Regulatory definitions and case law provide compelling indications that the display of food packaging for online retail falls into the definition of ‘labelling’ under the purview of FDA’s regulatory authority and that online viewing is a ‘customary condition of purchase’. The FDA may be able to use existing authority to engage in rulemaking to explicitly require the provision of required nutrition information to be highlighted for prominent and conspicuous viewing online. For the FTC, it is arguably the case that all missing or difficult to find required information in online food retail is an unfair and deceptive act or practice, based on consumers’ inability to identify this information, and the broad potential for harm and for consumers to choose differently if they had this information. The FDA and FTC could independently or in tandem engage in enforcement actions or issue guidance documents to ensure online food retail meets current regulatory requirements for food labelling.

For online retailers wishing to accept SNAP, the USDA’s authority may be even more compelling due to the risk of losing SNAP contracts. Congress’s allowance for the USDA to regulate SNAP retail food establishments indicates that the USDA could amend its regulations to require retail platforms that accept SNAP benefits ensure all required labelling is immediately visible, and conspicuously and legibly displayed. Current SNAP retailers include all of the largest food retailers in the country, including the major players online. Such requirements would benefit SNAP participants and the broader community of consumers shopping for food online^(42)^.

Whether these federal agencies will take meaningful action, or an act of Congress will be needed, remains to be seen. Congress could legislate requirements that online sale of food products must disclose all required information legibly, prominently and conspicuously. There is precedent for Congress to expand the application of labelling requirements to additional entities. In 2010, Congress extended FDA’s food labelling authority to include overseeing the provision of nutrition information by chain restaurants^(12)^. Congress can likewise amend the definition of labelling for the FDA to explicitly include online food sales. In addition, Congress could grant the FTC explicit authority, through Administrative Procedures Act rulemaking, to address online sale of food products or amend definitions related to SNAP to require online SNAP retailers disclose all required nutritional information.

Limitations of this study include that we examined a limited number of food product categories and foods, because the online coding portion of the research did not intend to be comprehensive. We, thus, may have missed additional deficiencies or strengths (e.g. disclosure of nutrition information when not required^(3)^) in the provision of nutritional information by online retailers. Moreover, we did not examine smaller, regional or local online retailers, which may have garnered different findings. Nonetheless, we did find information was not consistently available, and when available, it was not always conspicuous or readily legible across ten major products sold by the nine largest online retailers in the US, informing and supporting the need to examine federal regulatory authority to address these inconsistencies. This research did not identify which entity in the supply chain created product pages used for coding. The legal research did not examine state options to address the issues identified, such as options for the State Attorneys General to bring litigation^(43)^, state retail requirements or state regulation of online disclosure requirements. Moreover, this research did not evaluate unregulated labelling strategies such as interpretive labels voluntarily provided by manufacturers on the front of packages^(44)^. These are additional areas ripe for future research.

## Conclusion

The failure of online food retailers to consistently disclose required information may implicate health and safety concerns for consumers who depend on it, as in the case of allergens, Na or sugar; and others who may benefit from its provision. In the absence of uniform requirements, retailers may obscure nutrition information or highlight health or nutrition claims for less healthful products. Moreover, retailers’ new ability to track and target individual experiences means that the information could be provided to or withheld from shoppers to promote specific brands. Labelling requirements are intended to protect consumers who ‘are largely unable to protect themselves^(23)^’. This is even more salient for online sales where consumers cannot directly inspect products and retailers can decide which products to display, in what order, and with what accompanying advertisements. Therefore, at a minimum, the entire required nutritional information panel should be made conspicuously and immediately visible and legible under ordinary purchase conditions online.
